# Remarks on the Influence of Hysteria, in Modifying the Symptoms of Febrile Diseases

**Published:** 1831

**Authors:** James C. Finley


### Art. VIII.—*Remarks on the Influence of Hysteria in modifying the Symptoms of Febrile Disease.* By James C. Finley, M. D.

Most physicians, of much reading or practice, are familliar with the varied forms of hysteria, and the unpleasant symptoms which it often imparts to other diseases, when they attack constitutions pre-occupied by it. Every variety of inflammatory and nervous disease is occasionally imitated by it, and the vital organs, the heart, the lungs, and the brain, are sometimes so seriously affected as to give countenance to a treatment, so active as to occasion very serious injury.

At the commencement of febrile diseases the symptoms of hysteria so nearly coincide with many of the general symptoms of inflammatory action, as very frequently to escape detection; in the latter stages they may generally be known by the peculiar symptoms of nervous derangement which characterise this affection: in both stages they impart a degree of severity to the symptoms which would indicate a much greater degree of danger than really exists. The affection of the nervous system is manifested by delirium, stupor, convulsions, trembling or twitching of the muscles; and, when the inflammatory symptoms have been somewhat subdued, by that peculiar irritability, restlesness, and anxiety of mind, with the disposition to shed tears, and that alternate elevation and depression of spirits, which accompany this disease. The respiration is affected by oppression, laborious breathing, and deep sighing. The action of the heart is variously affected, and the pulse is frequently very much accelerated.

My attention was first drawn to this subject by observing some cases of autumnal fever, which occurred in patients laboring under derangement of the menstrual function, to be accompanied with many unusual symptoms. As several physicians to whom I have mentioned these circumstances, appear not to have been aware of the influence of this derangement in modifying febrile action in a marked degree, I feel rather disposed to doubt the fidelity of my own observations, and proceed to present the following cases to the readers of the Journal, rather with a view of eliciting further observation, than as laying before them the results of extended observation.

A negro girl, in Jones Co., Georgia, 15 years of age, was taken with a chill on the 12th of Nov., 1825. The chill came on with the most furious mania; the patient singing, hallooing, uttering the most obscene conversation, and biting her own flesh with such an exertion of muscular strength as to require two men to confine her. I saw her for the first time on the next morning, and directed her to be actively purged with calomel and jalap. On the third day I was with her when the chill came on with the same symptoms as before. She called the names of every individual in the room, but had no other idea respecting them. The chill continued about an hour, and when the fever began to rise, I bled her from the jugular vein until she fainted. She awoke entirely composed, though with some degree of stupor. In the course of the afternoon she took calomel 12 grs. with tart. ant. gr. i. divided into five powders, which operated several times upon the bowels. When the fever returned on the afternoon of the next day, the calomel and tartar emetic combined were prescribed, and continued through the night, bringing away thick feces like tar. On the next morning the mouth was affected, medicines were discontinued, and there was no return of fever. Menstruation had commenced in this patient about two months before, but had been suppressed in consequence of her having been exposed with thin clothing, to the cold heavy dews, in picking out cotton very early in the morning.

October 11th, 1828, I saw a female aged 18, on the third day of her illness; she had for some time been laboring under obstructed menstruation. She appeared insensible to any impression on the senses, and was singing, as if under some fanatical delusion. Sinapisms had been applied to the extremities and stomach, and when the mustard began to draw, she imagined herself in a future world, receiving the reward of a mispent life. When the fever began to rise as the pulse was weak, I was unwilling to take much blood, although there were such decided evidences of determination to the head. A vein was opened in the arm, and about ten oz. of blood taken.

She became calm and replied to our questions, and complained of the darkness of the room. In ten or fifteen minutes her vision returned, and she became perfectly rational. She was purged actively with calomel and jalap, and on the next morning took Dover's powder and quinine to prevent the return of the fever. The fever did not return, and by the use of the customary remedies the menstrual functions having been restored, the patient slowly regained her health.

On the 5th of Oct. 1830, I was called to visit Maria B., a young woman about 22 years of age, who was represented to be ill of the autumnal fever. I saw her in the afternoon, with considerable fever, tongue coated, pulse small, weak, and quick, right cheek flushed, pain in the chest, principally under the sternum; tenderness in the epigastrium, and troublesome cough. Her mother represented that about a month before, she had been caught in the rain during her menstrual period, that this discharge had been suddenly suppressed, and that during her present illness, the discharge had not returned at the usual period. The fever had been gradually coming on, and that within the last three days, she had been confined to her bed.

On the next morning, during the remission, the symptoms of local inflammation, except the cough, being relieved, I gave the sulph. quinæ, with the effect of mitigating the fever in the afternoon; on the 8th, sulph. quinæ again given in the morning. In the afternoon the febrile symptoms more alarming. The debility continued to increase, and the remission to become more indistinct. On the 12th, she presented the following symptoms: skin hot, cheeks flushed, pulse 120, cough troublesome, mind anxious, respiration oppressed, and interrupted by deep sighing, subsultus tendinum. On the following morning, during the intermission of the fever, the twitching of the muscles, restlessness, and anxiety of mind continued, accompanied with great concern about her domestic affairs, and a disposition to weep when conversing about her situation, which led me to refer many of her unfavorable symptoms to the hysteria.

All active treatment was now laid aside, and the patient put upon alternative doses of the blue pill, with ipecacuanha and assafœtida, and bitter tonics in the intermission. Under this treatment the unpleasant symptoms left her, and she gradually recovered her health.

I have been induced to publish these cases under the impression that the facts were sufficiently striking to justify me in laying them before the profession. Whether they sustain the views I have taken, I must leave to those who have a more extended experience, to determine.

Sixteen oz. blood was taken from the arm, and she was actively purged. On the next day, after cupping over the chest and epigastrium, a blister was applied to the chest. In the afternoon the pain in the breast was relieved, but the cough was troublesome, and the febrile symptoms were little mitigated. Prescribed tar. emetic, grs. ij, sulph. soda oz. i. dissolved in six oz. water:—a table spoonful every hour or two, till the bowels were freely opened, which was followed after the operation of the medicine, by an opiate.

